# Typhoid Fever and Its Treatment*Read before the Palestine meeting of the East Texas Medico-Chirurgical Society.

**Published:** 1902-07

**Authors:** S. N. Aston

**Affiliations:** Jewett, Texas


For Texas Medical Journal.

### Typhoid Fever and Its Treatment.*

BY S. N. ASTON, M. D., JEWETT, TEXAS.

We have selected this subject for this paper because we know of no one with which the general practitioner has to cope of more importance both to the practitioner and to the patient. Important to the doctor, because his reputation as well as his own peace of mind depend upon his success; important to the patient, because his life depends upon the success of the doctor, and we hope by this means to get an expression from each member present on the subject and thereby all be instructed rather than convey any new idea ourself.

Much has been said and written on this subject from time to time, much of which was valuable and is still doing honor to the author as a basis for new developments; while much has been weighed in the balances and found wanting; and while especially the last quarter of a century has achieved much on the road to success in this direction, still we believe that success in the treatment of this dread disease is still in its incipiency, for each point gained strengthens our vantage ground and there is yet, we believe, much progress to be made.

As the etiology and pathology of typhoid fever, together with its relation to sanitation, has been accepted by pretty much all the profession, and a paper covering the whole subject would necessarily be too long, as well as uninteresting, we shall only attempt to treat of that part of the subject wherein we may differ, viz., the treatment, and we shall confine ourselves to the medical treatment as we have had no experience in its surgical treatment, and in fact we believe there would be but few indications for surgical interference had all cases the proper medical attention throughout their course.

No doubt many of you realize at what a great disadvantage those of us who have largely country practice are placed in treating typhoid fever. 1. In having for our assistants one or both of two very objectionable classes of nurses—one not being intelligent enough to follow your directions during your absence, the other being too intelligent (?) to follow them and making such changes as they deem necessary. 2. The surroundings of the ordinary country home are not calculated to give your patient the best advantage. 3. The doctor cannot see his patient oftener perhaps than from twenty-four to forty-eight hours, and must leave him for that length of time at the mercy of inexperienced and often illiterate nurses, and enter the house on his return with fear and trembling, to ascertain whether his patient is yet alive; but these cases, like those under more favorable circumstances, must be entered on the doctor's record sheet, and I doubt not that being placed at such disadvantages we often get lessons that we might not otherwise.

In treating typhoid fever we would start out with the proposition

*Read before the Palestine meeting of the East Texas Medico-Chirurgical Society.

that, while in a sense it is a self-limited disease, as most authors claim, running its course and stopping if the patient holds out, but by the proper treatment it can be limited in its course, or, in other words, the length of time a case may run depends largely on the kind of treatment it gets. The early treatment of typhoid fever depends upon the condition of the patient when he receives the attack. If in a malarial district we have found that contrary to the typical course they very often take the form of a malarial attack, so much so that you are unable to make your diagnosis for several days, perhaps having distinct chills followed by high fever with all other symptoms of malaria, and after administering the anti-malarial treatment, which consists of mercury and quinine for two or three days, these symptoms will subside and the case take on the regular typhoid appearance, forcing us to the conclusion that typhoid fever is often complicated with malaria, notwithstanding the opinion of some good authors that there is no such disease as typho-malarial fever, hence we have adopted the rule in such cases of following the anti-malarial treatment for the first two or three days until we can make a positive diagnosis. We then leave off the quinine and antipyretics and endeavor to meet the indications as they arise, and right here we would say that in uncomplicated cases we believe there are but three main indications to be met: 1. Control the temperature. 2. Keep the secretions of the body active. 3. Nourish the patient.

To meet the first indication we know of nothing that will in any way compare with cold water in reducing temperature, quieting the nervous symptoms and producing quiet and restful sleep, not depressing the patient, but rather stimulating him. By the use of cold water, we mean applied externally, by whatever method is expedient at a temperature and in quantities to have effect and not stop short of the effect.

If you are situated so that you can use the complete bath, so much the better; but if not, then the cold pack is next best, and lastly the sponge bath. And whichever of the above methods is adopted there should also be an ice cap applied or cold water poured on the head and the latter seems to have the best effect in quieting the nervous symptoms.

If you have to adopt the tepid bath at first on account of the nervous condition of the patient gradually cool it down to at least the temperature of well water, or about 70° F.; and if it requires bathing for twenty or thirty minutes to get the effect, then bathe him that long. We use as much as ten or fifteen buckets of water at a time to pour on head, holding the vessel two or three feet above head, keeping it up until patient becomes quiet. We think that doctors often fail to get good results from the use of cold water because they are not persistent; and, again, they often leave word with whomsoever may be acting as nurse to bathe or sponge patient if fever gets high, and come back to find that it has not had the desired effect, instead of pulling off his coat and seeing that the patient gets the bath while he is there. We bathe patient if temperature gets above 103° F. or lower, or when very nervous, and continue until temperature is reduced to 102° or lower, or until nervous symptoms abate.

To meet the second indication: to keep kidneys active, the intestinal tract cleansed, the glandular secretions of the body stimulated, and in every way relieve the system of as much of the toxic accumulation as possible, we find the mild chloride of mercury by far the best remedy we have ever used, and we do not mean by the use of mercury one-fourth grain every four hours for three or four doses as some have recommended, but to give it for its effect. We ordinarily begin with two grains in combination with bicarbonate of soda every two hours, and continue until we get several copious discharges, usually requiring from ten to twelve grains (for in this abnormal condition of the intestinal secretions you must remember the absorptive powers are subnormal, hence you will not get full effects of the mercury). We then reduce the dose or lengthen the interval between, keeping it up until the secretions of the body are well aroused and contents of the intestinal tract removed. This condition is indicated by moist tongue and absence of tympanitis and tenderness in the bowels. We repeat this course as often as the tongue becomes dry and the other symptoms aggravated which will be sure to accompany the dry tongue.

This condition will begin to return every second or third day usually, so that our patient ordinarily gets from two to three courses of the mild chloride of mercury during each week, averaging for each course about ten grains. We keep this up until convalescence is well established, and we will say here where the case has come under our care during the early part of its course we have never had a troublesome diarrhea nor a hemorrhage from the bowels, nor does the fact of the hemorrhage existing when we see them later in the course of the disease contraindicate the use of mercury. Our reason for pursuing this course is this: In typhoid fever we have Pyer's patches and the solitary glands of the small and large intestines inflamed and congested, also liver and spleen enlarged and congested and performing their functions abnormally, while the contents of the intestines are laden with typhoid bacilli, which by their presence and the toxins excreted by them and in the absence of the normal secretions of the intestinal glands and the bile secreted by the liver, which is the natural intestinal antiseptic, a fermentation takes place which furnishes a still more favorable breeding ground for the bacilli, not only irritating the already inflamed glands and causing distension of the intestinal wall, but causing by its absorption new infection and increase of temperature and the nervous symptoms so characteristic of this disease; also producing diarrhea and the hemorrhage which so often ends the anxious scene.

Now, the mild chloride of mercury possesses many of the qualities to meet these indications. 1. It is one of the best diuretics we have, increasing the toxic contents of the urine. 2. It is an antiseptic. 3. It is antiphlogistic in its effect. 4. It excites the secretions of the body and especially that of the liver. 5. It is an alterative and, lastly, it is a mild purgative, cleansing the alimentary tract of all irritating matter. We claim by the early and continuous use of this drug in sufficient quantities to have effect we do often shorten the course of the disease, for in the beginning we may have only a few of the glands of the intestines infected by the bacilli, and by the combined effects of the mercury in moving off the contents of the intestines, which is composed of a fermenting mass laden with the bacilli and their toxines, and the depleting effect on the glands already effected, together with its action on the liver, causing a normal flow of bile into the intestine, we say by these effects we prevent the spread of infection to other glands and promote healing of those already affected; or, to make a comparison, we would treat the intestinal tract in this condition as we would an infested cavity anywhere in the body, and the effects will be similar. In that case we would by all means evacuate the cavity, sterilize and drain to prevent absorption of the contents, which would cause not only a continuation of the process, but also constitutional effects, just so you want by the best means at your command to remove contents of the intestines and produce constant and thorough drainage, at the same time as best you can sterilize the tract.

To meet the third indication: that of nourishing the patient. We have no certain diet or set of diets to prescribe, but suit the diet largely to the taste and condition of the patient, there being a great many diets which are equally good and harmless. We usually diversify the diet, having, if any difference, a preference for a good quality of fresh buttermilk. We make it of first importance that the nourishment should be of easy assimulation, having but little residue, and, secondly, that it should be given in sufficient quantity to support the patient, and at regular intervals, giving sufficient time for digestion to be complete between each administration; hence we direct nourishment to be given every three or four hours, giving at each time from two-thirds to a full glass of milk or its equivalent, and keeping up liquid nourishment for eight or ten days after fever has subsided.

Now, in connection with the above, which is our basis of treatment, we of course use other agents to meet certain conditions. Where we have a fetid diarrhea we give sulpho-carbolate of zinc; where tongue is very dry and bowels tympanetic and tender we give spirits of turpentine in 5-6 drop doses, at the same applying hot turpentine stupes to the abdomen. In connection with and in the intervals between the mercury we often give the Woodbridge Typhoid Fever Tablet (No. 2), and when needed we give some good digestant; also a diuretic, and direct nurse to give patient plenty of cold water to drink. Especially after second week we put patient on the following mixture:

℞ Pot. chlorate .......................... ʒi.

Tr. nux vomica.......................... ʒivss.

Fowler's sol. .......................... ʒii.

Spts. nitros ether.......................... ℥i.

Aqua dist. qs.......................... ℥iv.

M. Sig. Teaspoonful in water every four hours during the day, this to be kept up until convalescence is well advanced.

We have followed the above plan of treatment in typhoid fever for five or six years, having in that time a fair share of patients for a general practitioner, and so far have never lost a case; neither have we had, where patient came under our care during early part of course, a troublesome diarrhea or hemorrhage from the bowels, hence we give it with confidence as a pactical fact and not a theory only.

Were it not for prolonging this already lengthy paper we would report two or three cases which we have treated in the last few weeks in connection with our co-worker, Dr. Spruiell, and which represented the graver type of the disease, the result in each case being entirely satisfactory to us. We hope to have an expression from each one present on this subject.

